# The effect of high temperature and humidity on milk yield in Ankole and crossbred cows

**DOI:** 10.1007/s11250-022-03092-z

**Published:** 2022-02-03

**Authors:** Yvan Bienvenu Niyonzima, Erling Strandberg, Claire D’Andre Hirwa, Maximilian Manzi, Martin Ntawubizi, Lotta Rydhmer

**Affiliations:** 1 School of Veterinary Medicine, University of Rwanda-Nyagatare Campus, P.O. Box 57, Nyagatare, Rwanda; 2 Department of Animal Breeding and Genetics, Swedish University of Agricultural Sciences, Box 7023, 75007 Uppsala, Sweden; 3 Rwanda Agriculture and Animal Resources Development Board, P.O. Box 5016, Rubona, Huye District, Rwanda

**Keywords:** Heat stress, THI, Dairy cattle, Ankole, Crossbred, Milk production

## Abstract

Tropical regions are characterized by high temperature and humidity across the year. At high values of temperature humidity index (THI), there is a risk of heat stress leading to lower milk yield. The objective of this study was to describe the effect of season and the effect of maximum daily THI on milk yield of that day in purebred Ankole and Ankole-Friesian, Ankole-Jersey and Ankole-Sahiwal crosses in a tropical climate. In total, 53,730 records of daily milk yield from 183 cows in Rwanda were analyzed. The results showed that THI had a negative effect on daily milk yield above a threshold, but the effect was small (− 0.11 kg milk/THI unit at most). Purebred Ankole cows had the lowest daily milk yield and the lowest threshold (THI mean 66), as compared to the crossbreds (THI mean 68–69). Ankole-Friesian had a steeper decline in daily milk yield above the threshold than Ankole. The crossbreds, especially Ankole-Friesian, had higher daily milk yield than purebred Ankole also at very high THI. The results indicate some differences between breed groups in the way of coping with a hot and humid climate and raise questions about dairy cows’ adaptation to such a climate.

## Introduction

Dairy cattle play an important role for food security in Rwanda (Ter Steeg 2019) and there is a clear political will to increase dairy production (Mugabo et al. 2019). The indigenous Ankole breed is characterized by a high ability to cope with harsh environments, but the milk yield is low (Wurzinger et al. 2014; Manzi et al. 2020). Crossbreeding has been part of the government policy in order to increase milk and meat production, and state-subsidized artificial insemination campaigns have resulted in hundreds of thousands of crossbred cows in Rwanda (Manzi et al. 2018). Crosses between Ankole and Friesian (Holstein), Jersey and Sahiwal are common.

There is a genetic antagonism between milk production level and heat tolerance (Carabaño et al. 2014) explained by the high metabolic heat production associated with milk production (Baumgard and Rhoads 2012). The climate in Rwanda is characterized by rather high temperature and humidity during large parts of the year. The year consists of four seasons. The two rain seasons are with short rains (season SRS) falling between September and December, and long rains (season LRS) extending from March through May. The two dry seasons extend from June to August (LDS) and January to February (SDS). Milk production of purebred Ankole and crossbreds in Rwanda has been studied by Manzi et al. (2020), who showed a significant effect of calving season on average daily milk yield. It was not possible to separate the direct effect of the weather from the effect on feed availability and quality in that study, but it is plausible that the effect of season was associated with high temperature and humidity causing heat stress.

Temperature humidity index (THI), which combines temperature and relative humidity, is often used in studies of heat stress (Dash et al. 2016). Many studies have shown the risk of reduced production and animal welfare due to heat stress in periods with high THI (reviewed by Hahn et al. 2009). In Sub-Saharan Africa, drought and dry seasons are becoming more frequent and longer in duration, resulting in heat stress for dairy cattle (Kekana et al. 2018). The ongoing climate changes may increase the negative effect of high THI on milk yield in Rwanda and many other countries.

Our aim was to study the effect of THI on milk yield in purebred Ankole cows and in crossbreds between Ankole and Friesian (Holstein), Jersey and Sahiwal. We hypothesized that purebred Ankole cows and Ankole x Sahiwal crosses are less affected by high THI than Ankole crosses with the exotic breeds Friesian and Jersey.

## Material and methods

### Animal management and milk records

The animals were raised and kept on natural pasture without supplementary feeding. They grazed in large paddocks and only young calves, cows calving and sick cows were housed. Water was provided twice daily. In the first week following calving, calves were allowed to suckle freely. Thereafter, partial milking was practiced and calves were separated from their mothers outside milking hours. At milking, the calves were allowed to suckle their mothers to stimulate milk let-down and after milking they were allowed to suckle the residual milk. The calves were weaned when the cows were dried off. The cows were hand milked in the morning (from six to seven) and in the evening (from five to six). Date, cow ID number and milk yield (in litre, L) were recorded on paper at every milking. Morning and evening yields were added to a daily milk yield (DMY). This milk yield describes the amount of milk excluding the milk intake of the calf. Calvings were spread over the year. In a previous study of these cows, the average lactation length was reported to be 256 days across all breed groups, and breed group was not significant for lactation length (Manzi et al. 2020).

Milk yield data from two research stations belonging to Rwanda Agriculture and Animal Resources Development Board were used. When both morning and evening milk yields were recorded as 0 L, the daily milk yield was regarded as a missing value (i.e. not 0 L). Two cows with only one milk record each were deleted from the data set. Thereafter, the Songa station had 41,017 daily milk yield (DMY) records from 143 cows and the Rubona station had 12,713 DMY records from 40 cows. Both stations are located in the Huye District (Songa 2° 24′ S, 29° 46′ E; Rubona 2° 30′ S, 30° 25′ E). In total, the data set included 53,730 DMY records from June 2014 to May 2017.

### Weather records

Weather data were obtained from the Rwanda Meteorology Agency and consisted of daily weather information from a weather station located at Rubona (2° 08′ S, 29° 58′ E; altitude 1742 m) 15 km from the Songa farm and 3 km from the Rubona farm. The weather information included the minimum and maximum (Maxtemp) daily ambient temperature of each day (24 h) and the average relative humidity (hereafter humidity) of each day. Humidity was calculated as the average of records from every tenth minute during 24 h. The minimum and maximum temperature records were used to calculate an average temperature value for each day (Meantemp).

Daily records of Maxtemp and humidity were used to calculate the daily maximum THI (THImax) using the formula from National Research Council (1971). This formula is according to a review by Herbut et al. (2018) one of the most used THI formulas in studies of heat stress and dairy cows. Temperature in degrees Celsius and humidity in percent are inputs in the formula: *THImax* = (1.8 × *Maxtemp* + 32)—(0.55–0.0055 × *humidity*) × (1.8 × *Maxtemp*-26).

The daily mean THI (THImean) was calculated in the same way as THImax, with Meantemp instead of Maxtemp. To illustrate the meaning of THI, different combinations of temperature and humidity resulting in a THI value equal to 75 or 76 are presented in Table 1.

**Table 1:** Temperature and relative humidity range when THI is equal to 75 or 76

	THI = 75		THI = 76	
Temperature, °C	Min humidity, %	Max humidity, %	Min humidity, %	Max humidity, %
24	93	100		
25	77	85	86	95
26	63	71	72	79
27	51	58	59	67
28	42	48	49	56
29	33	39	40	46
30	26	31	32	38
31	19	24	25	31
32	14	18	19	24
33	9	13	14	18
34	4	8	9	13
35	0	4	5	9
36			1	4
37			0	1

### Statistical analysis

The total number of daily milk yield (DMY) records used in the analysis was 53,730 records, the largest part of which was from purebred Ankole cows. In total, 183 cows were included in the study (Table 2).

**Table 2:** Number of included cows (*N*), daily milk yield (DMY) records per cow (average, minimum, maximum), total number of records and average DMY in L per cow (least squares mean from model 1), per breed group

		No. records per cow			Total	Average
Breed group	*N*	Average	Minimum	Maximum	No. records	DMY^1^
AA	78	311	4	934	24,248	2.00^a^
AF	41	293	16	834	12,022	4.01^b^
AJ	36	322	10	673	11,601	3.36^c^
AS	28	209	14	734	5859	3.35^c^

*AA*, purebred Ankole; *AF*, Ankole × Friesian; *AJ*, Ankole × Jersey; *AS*, Ankole × Sahiwal

^1^Mean values with different superscripts are significantly different (*p* < 0.0001)

**Fig. 1:**
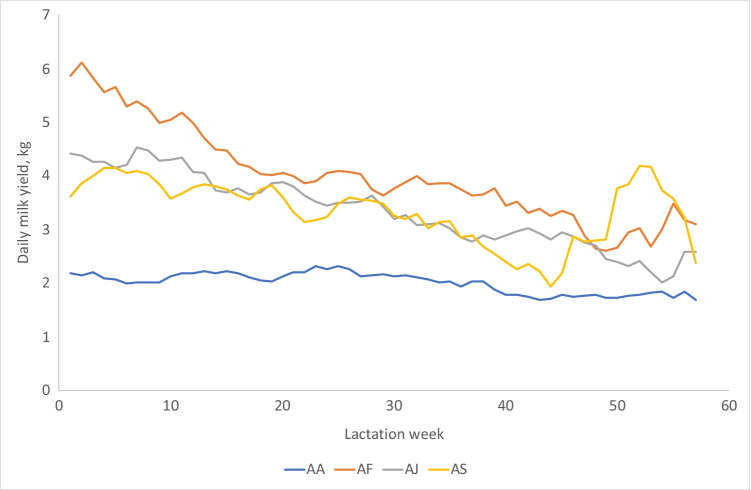
Daily milk yield (excluding the calf’s milk intake) as a function of lactation week for purebred Ankole cows (AA), crossbred Ankole × Friesian (AF), Ankole × Jersey (AJ) and Ankole × Sahiwal (AS) cows

The statistical analysis was performed with SAS (SAS Institute Inc., 2017). Three models were applied to analyze DMY. All models included four fixed effects not related to thermal load: station, breed group, parity and lactation stage. They are called NTE (not thermal load) in the models below.

$$NTL={stn}_{i}+{bg}_{j}+{par}_{k}+{stage}_{l\left(j\right)}$$

where *stn*_*i*_ is the fixed effect of station *i* (2 classes, Rubona and Songa), *bg*_*j*_ is the fixed effect of breed group (4 classes), *par*_*k*_ is the fixed effect of parity (7 classes: 1, 2, …, 5, 6–7, 8–11)), *stage*_*l*_ is the fixed effect of lactation stage (lactation week, but days before day 7 was included in week from day 7 to 13 and weeks above 57 were included in week 57, Fig. 1) nested within breed group. The breed groups were purebred Ankole (AA) and F1 crossbreds between Ankole and Friesian (AF), Ankole and Jersey (AJ) and Ankole and Sahiwal (AS). Five cows had no record on parity number and they were assumed to be in parity 3 (the most common parity number). Days in milk (DIM) was unknown for 14% of observations because calving dates were unknown; for these the first daily milk yield was set to day 7 (i.e. 1st lactation week). In the data with known DIM, 82% had their first DIM before day 14; thus, the applied approximation should work well for most cows.

Model 1 was used to estimate the significance of main effects:

1 $${y}_{ijklmno}=\mu +NTL+{seas}_{m}+{cow}_{n}+{e}_{ijklmno}$$

where *seas*_*m*_ is the fixed effect of season of the day milk was recorded (4 classes: SDS, LRS, LDS, SRS).

In model 2, instead of season, THImax or THImean values rounded to integers (rTHImax, rTHImean) were included as a fixed class variable, across [2a] and within breed groups[2b]:

2a $${y}_{ijklmno}=\mu +NTL+{rTHI}_{m}+{cow}_{n}+{e}_{ijklmno}$$

2b $${y}_{ijklmno}=\mu +NTL+{rTHI}_{m(j)}+{cow}_{n}+{e}_{ijklmno}$$

**Fig. 2:**
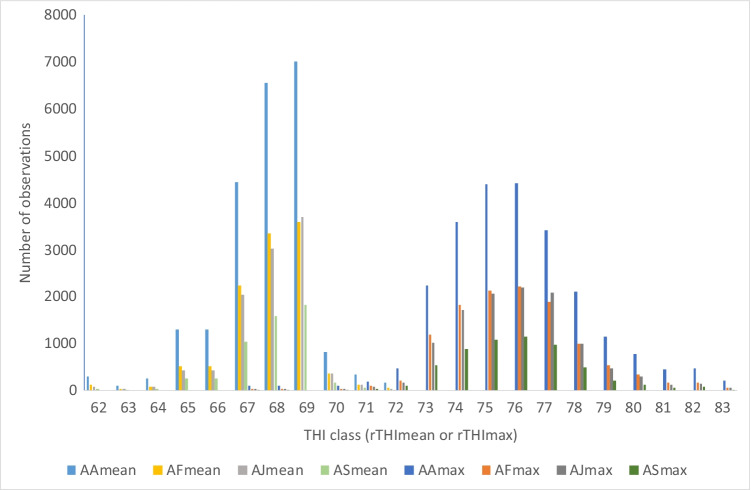
Number of observations in various classes for average THI value (rTHImean, to the left) and maximum THI value (rTHImax, to the right) for each breed group

The rTHImax classes 63 and 65 (64 and 66 were missing) were combined with class 67, 69 was combined with 68 and classes 84–85 were combined with class 83, because there were fewer than 10 observations for at least one breed group. The number of observations included in each rTHImax class ranged from 69 (for rTHImax 69) to 9992 (for rTHImax 76). Similarly, the lowest rTHImean class was 62 (which included class 57 and 61) and the highest was 72 (included also class 74). The number of observations included in each rTHImean class ranged from 198 (for rTHImean 63) to 16,129 (for rTHImean 68). The distribution of number of observations is shown in Fig. 2.

**Fig. 3:**
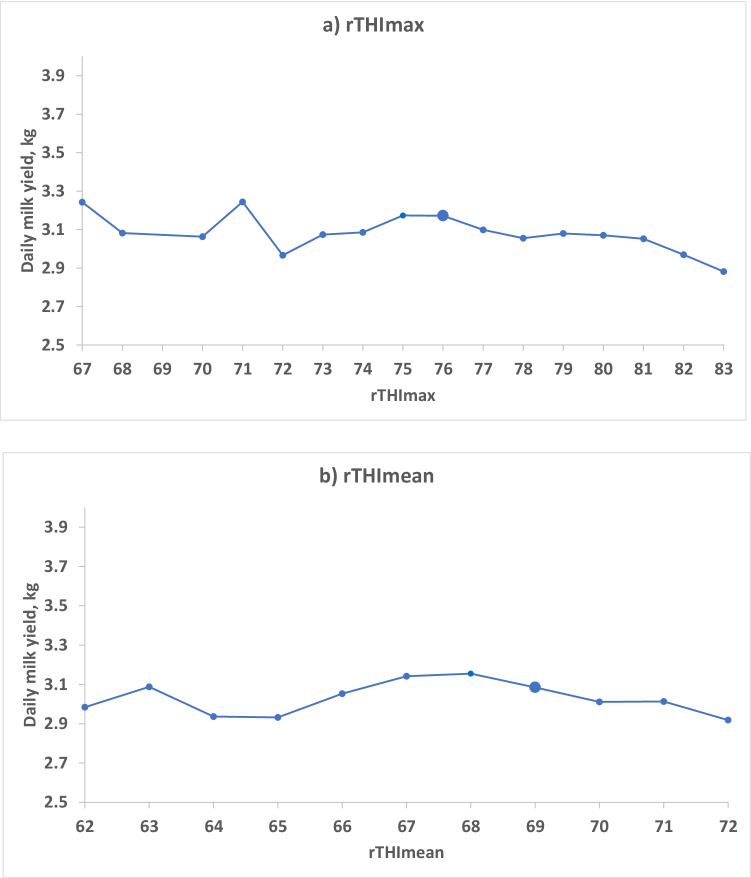
The effect of the maximum THI value (rTHImax, Fig. 3a) and the effect of the average THI value (rTHImean, Fig. 3b) during the day on the milk yield of that day, illustrated with least squares mean values from model 2a. *N* = 53,730 milk yield records from 183 cows. The designated threshold is shown with a larger marker

**Fig. 4:**
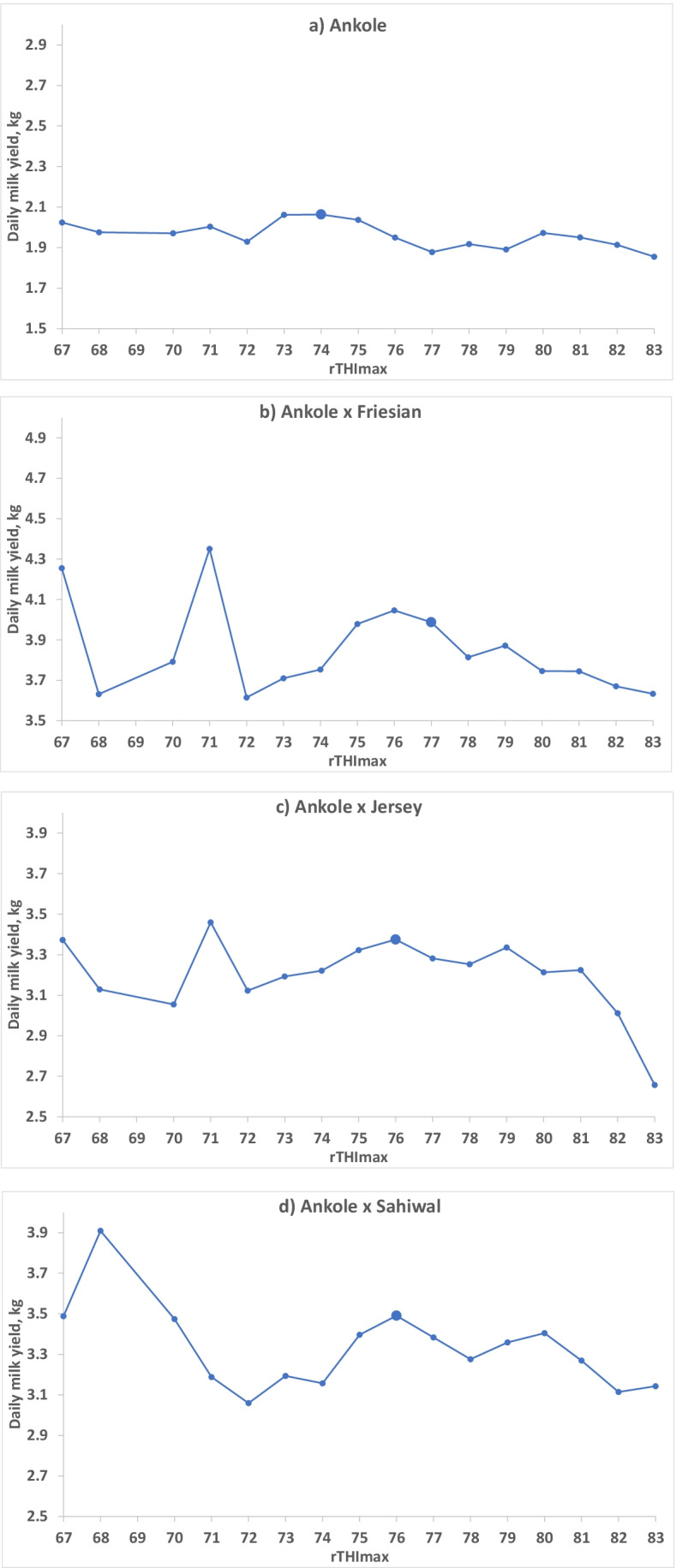
Daily milk yield (least squares means from model 2b) at different rounded daily maximum THI values (rTHImax) for **a** purebred Ankole cows (AA; *N* = 24,248 milk yield records from 78 cows) and **b** crossbred Ankole × Friesian (AF; *N* = 12,022 milk yield records from 41 cows), **c** Ankole × Jersey (AJ; *N* = 11,601 milk yield records from 36 cows) and **d** Ankole × Sahiwal (AS; *N* = 5,859 milk yield records from 28 cows). The designated threshold is shown with a larger marker

An approximate threshold level of rTHImax or rTHImean, above which milk yield decreased with increasing THI, was determined as the rTHImax or rTHImean value with the highest average milk yield before a clear downward trend could be seen, using least squares means from model 2, both across (model 2a, Fig. 3) and within breed groups (model 2b, Fig. 4).

Model 3 was identical to model 2 except that a fixed regression of DMY on positive deviations of THImax or THImean from the threshold was used instead of the class effect of rTHImax or rTHImean. This model was used to estimate a regression both across [3a] and within breed groups [3b].

3a $${y}_{ijklmno}=\mu +NTL+{b THIdev}_{m}+{cow}_{n}+{e}_{ijklmno}$$

3b $${y}_{ijklmno}=\mu +NTL+{{b}_{j} THIdev}_{m}+{cow}_{n}+{e}_{ijklmno}$$

The regressor (*THIdev*) was 0 at and below the assumed threshold. Several values of the threshold were tested in model 3 based on the results from model 2 and the threshold from the model with the best (lowest) Akaike Information Criterion (AIC) was chosen, and regression estimates from that model are presented.

## Results

According to the weather station data, THImax ranged from 63.3 to 84.6, with an average of 75.8 and THImean ranged from 57.3 to 73.8, with an average of 67.4. During SDS, which was the hottest season, all days had a THImax above 73. Average temperature, relative humidity and THImean and THImax for the four different seasons during the studied years are presented in Table 3.

**Table 3:** Number of weather records, averages of temperature (Meantemp), relative humidity, THI mean value (THImean) and average THI maximum value (THImax), together with proportion of days with THImax > 75 and maximum THImax value (max THImax), for each season

Season	No. weather records (days)	Meantemp	Humidity	THImean	THImax	Days with THImax > 75, %	Max THImax
SDS	178	21.0	73.1	68.0	76.8	85	82.3
LRS	257	20.8	78.3	68.0	76.8	86	81.9
LDS	268	20.8	56.5	66.6	74.6	32	83.3
SRS	366	20.6	75.1	67.6	76.5	76	84.6

*SDS*, short dry season (Jan–Feb); *LRS*, long rain season (Mar–May); *LDS*, long dry season (Jun–Aug); *SRS*, short rain season (Sep–Dec)

All fixed factors in model 1 (station, breed group, parity, season and lactation stage nested within breed group) were significant (station *p* = 0.0015, other effects *p* < 0.0001). Average daily milk yield excluding the milk intake of the calf (DMY) for Ankole and the crossbreds are shown in Table 2. The positive effect of crossbreeding on daily milk yield was substantial; Ankole had lower milk yield than all crossbreeds (*p* < 0.0001). DMY increased with parity (lactation) until parity 5 (Table 4). Season had a significant effect on DMY, and DMY was highest during LRS and lowest during SRS (Table 5). The lactation curves for the breed groups are shown in Fig. 1. Purebred Ankole had a flat lactation curve.

**Table 4:** Number of records and average daily milk yield (DMY, in L, least squares means from model 1), per parity

Parity	No. records	Ave. DMY^1^
1	9688	2.78^a^
2	11,255	2.76^a^
3	15,722	3.10^b^
4	10,182	3.27^c^
5	3726	3.67^d^
6–7	1646	3.08^b^
8–11	1511	3.60^abcd^

6–7 = parity 6 and 7; 8–11 = parity 8, 9, 10 and 11

^1^Mean values with different superscripts are significantly different (*p* < 0.05)

**Table 5:** Number of records and average daily milk yield (DMY, in L, least squares means from model 1), per season

Season	No. records	Ave. DMY^1^
SDS	8363	3.31^a^
LRS	11,198	3.49^b^
LDS	15,099	3.00^c^
SRS	19,070	2.92^d^

*SDS*, short dry season (Jan–Feb); *LRS*, long rain season (Mar–May); *LDS*, long dry season (Jun–Aug); *SRS*, short rain season (Sep–Dec)

^1^Mean values with different superscripts are significantly different (*p* < 0.0001)

Least squares mean from model 2a of DMY for rTHImax and rTHImean values across all breed groups are shown in Fig. 3. The effect of rTHImax on daily milk yield was highly significant (*p* < 0.0001). DMY decreases when rTHImax is above the threshold of 76. DMY was similar for rTHI = 75, but the best threshold was estimated at 76 in accordance with the AIC of model 3a (167,718.4) (see Table 1 for an illustration of temperature and humidity values when THI is equal to 75 or 76). Values at lower rTHImax fluctuated more and there were relatively fewer observations up to around rTHImax of 73 (Fig. 2). Figure 3b is a corresponding plot of rTHImean values from 62 to 72. The effect of rTHImean on daily milk yield was also significant (model 2a, *p* < 0.001), with an optimal threshold value of 69 (68 had almost the same AIC 167,737.8 vs 167,737.9 in model 3a).

**Fig. 5:**
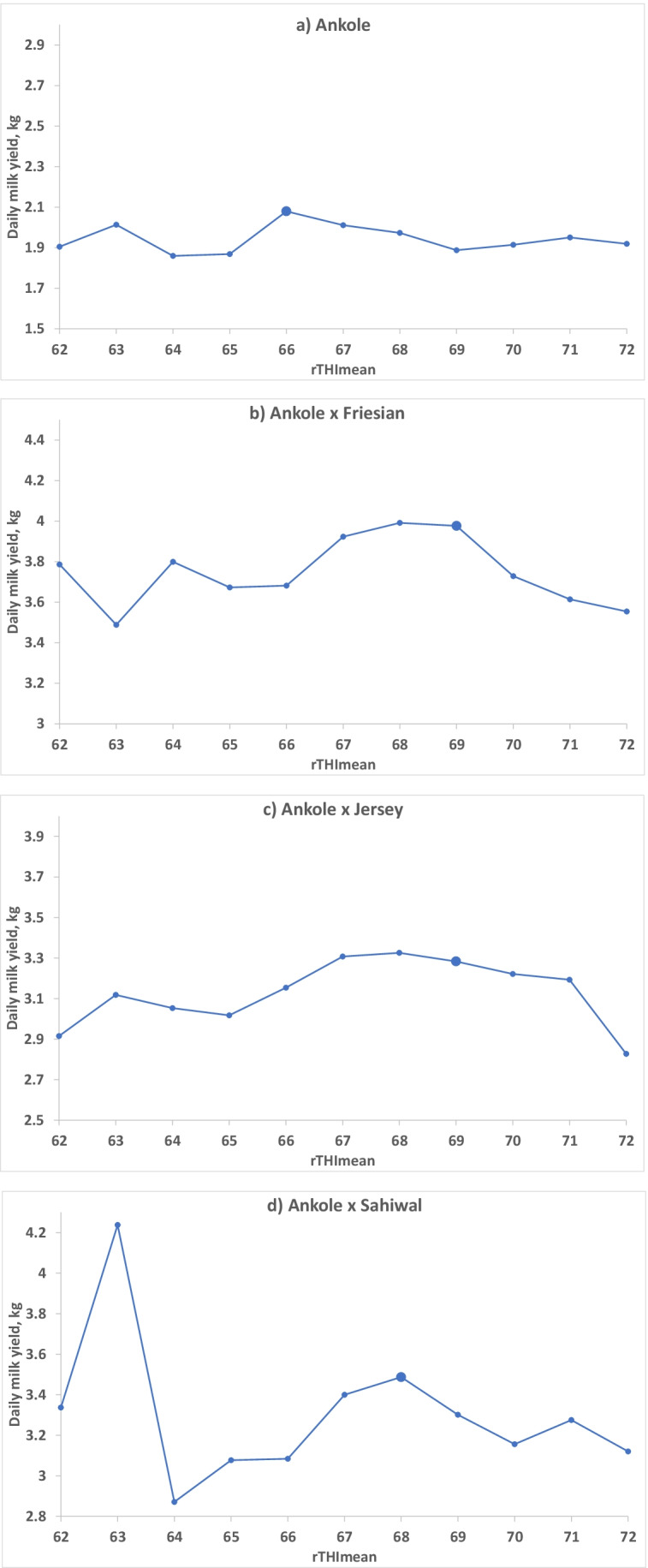
Daily milk yield (least squares means from model 2b) at different rounded daily mean THI values (rTHImean) for **a** purebred Ankole cows (AA; *N* = 24,248 milk yield records from 78 cows) and **b** crossbred Ankole × Friesian (AF; *N* = 12,022 milk yield records from 41 cows), **c** Ankole × Jersey (AJ; *N* = 11,601 milk yield records from 36 cows) and **d** Ankole × Sahiwal (AS; *N* = 5,859 milk yield records from 28 cows). The designated threshold is shown with a larger marker

The value for rTHImax, above which milk yield decreased, was estimated for each breed group separately by testing the potential values based on the least squares means using model 2b (Fig. 4). In Ankole, milk yield started to decrease when THImax was higher than 74 (best AIC with model 3; values 73–75 were tested). The optimal threshold values for AF, AJ and AS were 77 (76–77 tested), 76 and 76 (only value tested), respectively. The corresponding thresholds for rTHImean were found to be 66, 69 (68 and 69 tested), 69 (67–69 tested) and 68 for AA, AF, AJ and AS, respectively (Fig. 5, Table 6). The AIC for the best model for THImax was 167,707.7 and the best for THImean was 167,729.3, indicating that THImax was explaining more of the variation in milk yield. The corresponding AIC for the models applied across breed groups were 167,718.4 and 167,737.8, respectively, so the models with regressions nested within breed group were better.

**Table 6:** Threshold values and change in daily milk yield (DMY) with increasing THImax or THImean after threshold (*b*; *b*-value expressed in L milk/day/THI) from model 3, per breed group

Breed group	THImax		THImean	
	Threshold	*b*	Threshold	*b*
All	76	− 0.023***	69	− 0.060***
AA	74	− 0.025***	66	− 0.030***
AF	77	− 0.045***	69	− 0.107**
AJ	76	− 0.023**	69	− 0.084*
AS	76	− 0.010^ ns^	68	− 0.039^ ns^

*AA*, purebred Ankole; *AF*, Ankole × Friesian; *AJ*, Ankole × Jersey; *AS*, Ankole × Sahiwal

*p*-values for regression coefficients: *ns*, not significant; * < 0.05; ** < 0.01; *** < 0.0001

The *b*-values presented in Table 6 describe the effect of increasing THImax or THImean from the threshold upwards for purebred Ankole cows and the three crossbreds. As an example, increasing THImax with 10 units above the threshold is expected to decrease milk yield by 0.25 kg for purebred Ankole. All four breed groups had negative regression coefficients and AF had the steepest decline. The slopes for AS were not significantly different from zero.

## Discussion

High THI has a negative influence on daily milk yield according to many studies (Kadzere et al. 2002). For the cows from Rwanda in this study, the THImax threshold was estimated at 76 (from 74 for Ankole to 76–77 for crossbreds). This is below or close to the average THImax in all four seasons in the studied location. Thus, THImax is over the threshold many days of the year. However, for our data, it seems that there is very little influence of THI above the threshold on the milk yield. Arguably the 50% Ankole in the crosses is enough to make the cows rather resilient to high THI, but it would have been interesting to see the effect for purebred exotic breeds, i.e. Holstein–Friesian or Jersey animals, under the same climatic conditions. In general, heat stress has a larger negative effect on high-producing animals (Kadzere et al. 2002). Indeed, AF had the steepest decline, possibly indicating that Holstein–Friesian cattle are more affected by heat stress than the other breeds.

We do not know if the milk consumption of calves is affected by THI. Significant differences between Holstein–Friesian calves provided cooling or not were reported by Dado-Senn et al. (2020). The cooled calves had higher daily intake of milk replacer than the heat-stressed calves. If, say, the milk consumption decreased as a function of increased THI in our study, then the true, total decrease in milk yield of the cows would be larger than presented. For the comparison between breed groups, it should also be remembered that we do not know if the average daily milk intake of the calf differs between breed groups.

Milk yield was collected daily and associated with THI of the same day. Thus, we studied the acute stress of high temperature and humidity. With high THI during several days in a row, the possibility to dissipate body heat at night may be reduced. As a result, the increase in body temperature is accumulated from day to day during a heat wave, leading to significant declines in production (Kadzere et al. 2002). A complement to the effect of THImean could be the effect of THImin, which would reflect the conditions during nights. Another alternative could be to study the effect of average THImax or THImean during the week preceding each DMY record. In a preliminary analysis, we replaced THImax with THImax of the previous day, and the results were similar to the results reported here. Furthermore, most of the days had similar THImax (± 1 unit) as the previous day. Nevertheless, the effect of high THI during a range of days preceding the milk record could be interesting to analyze in future studies.

The effect of season is more than just THI. LDS has lower THImax and THImean than SDS but the average DMY is lower during LDS. It is well known that heat stress reduces voluntary feed intake (Kadzere et al. 2002), but feed intake on pasture can also be reduced due to feed shortage during dry periods. Heat stress in livestock is aggravated during feed restriction (Sejian et al. 2012). The cows in this study were on pasture and daily feed intake could not be recorded. It can, however, be assumed that the lower DMY during LDS was a consequence of feed shortage in combination with physiological and metabolic effects of heat stress. Cowley et al. (2015) studied Holstein–Friesian cows in climate chambers. Heat-stressed cows had lower voluntary feed intake and lower milk yield than control cows in a temperate climate. Cows in a temperate climate fed with the same amount of feed as the heat-stressed cows had lower milk yield than the control cows, but the difference was smaller and not significant. It would be interesting to study voluntary feed intake of different breed groups with and without heat stress, and also to study their reduction in milk yield under a feed restriction reflecting the pasture quantity and quality during LDS.

The overall THImax threshold of 76 in this study may seem high, but the cows have a very low DMY compared to many other studies, and there is an unfavourable correlation between production level and tolerance to heat stress (Kadzere et al. 2002). According to De Rensis et al. (2015), THI between 68 and 74 cause mild discomfort and problems, and for THI above 75, cows can show “noticeable decreases in performance”. In Mexico, Fernández et al. (2019) reported a THI threshold (based on monthly averages in temperature and relative humidity) at 73 for milk production of Holstein–Friesian cows with a production level around 30 kg per day. Ravagnolo et al. (2000) report a THI threshold at 72 for milk production of Holstein–Friesian cows with a production level around 26 kg per day. Differences in THI equations make it difficult to compare threshold values across studies, and it is also not always clear whether the presented THI value corresponds to THImax or THImean.

Estimates of the slopes of production decline vary across studies. For the cows with the highest DMY in this study, i.e. the AF cows, the *b*-value was − 0.045 for THImax and − 0.107 for THImean. In the study by Ravagnolo et al. (2000), the *b*-value was − 0.2 above a THI threshold at 72. Santana et al. (2016) studied Holstein–Friesian cows in Brazil where the average THI was 66. The *b*-value was − 0.25 for 1st lactation cows (DMY 30 kg/day) and − 0.47 for 3rd lactation cows (DMY 35 kg/day). Bouraoui et al. (2002) reported that Holstein–Friesian cows in a Mediterranean climate with an average DMY of around 18 kg/day had a *b*-value of − 0.41 above a THI threshold at 69. Bernabucci et al. (2014) used a THI average from 8 days before the day milk yield was recorded, in a study of Holstein–Friesian cows in Italy. The *b*-value was − 0.91 for 1st lactation cows (DMY 30 kg/day) and − 1.27 for 3rd lactation cows (DMY 35 kg/day). Again, the lower slopes in our study may be related to the low production level (DMY 2–4 kg/day).

Our hypothesis was that the breed Ankole, which is a native breed in the tropical environment of central Africa, would have a higher ability to cope with a hot climate, as compared to exotic breeds. Thus, we expected a higher THI threshold for purebred Ankole cows than for crossbreds with exotic breeds. This was not true; the THI threshold seemed to be slightly lower for purebred Ankole (THImax threshold 74) than for the crossbreds including exotic breeds (76–77). On the other hand, the effect of high THI tended to be less severe for Ankole than for Ankole-Friesian. This was shown as a lower slope in milk yield decrease above the threshold. Even though the decrease in DMY above the threshold was lower for purebred Ankole cows, their average milk yield was lower than the milk yield of Ankole-Friesian and other crossbreds also at very high THI values. It would have been interesting to have pure Holstein–Friesian, Jersey or Sahiwal cattle to compare with. Berman (2011), reviewing breed characteristics related to tropical climate tolerance, found no clear evidence that breeds evolved in a tropical environment have higher capacity for heat dissipation. According to Berman (2011), adaptation to a tropical climate “diminished climate-induced strain by decreasing milk production”.

Bos indicus breeds, such as Sahiwal and Gir, are considered to be better adapted to a tropical climate and less susceptible to heat stress, as compared to Bos taurus breeds (Santana et al. 2015). Accordingly, AS tended to have a higher threshold for THImax than the Bos taurus AA in our study. In Brazil, Gir cows with an average milk production around 13 kg/day had a THI threshold (average daily THI) for milk production at 69 (Santana et al. 2015). Above this threshold, daily milk yield decreased with 0.09 kg/day for each THI unit. The genetic trend for milk yield was positive, but in parallel with this favourable trend, the difference in breeding values for milk yield estimated at THI = 58 and THI = 81 increased. According to the authors, these results should serve as a warning for breeding programs of Gir cattle. Likewise, if Ankole cattle are selected for increased milk yield, it seems wise to monitor the genetic trend in tolerance to heat stress.

The rejection of our hypothesis made us reflect on the meaning of adaptation to hot climate for dairy cows. Is the highly adapted cow a cow that continues to produce the same DMY for as long as possible, in spite of a rising THI? What happens with that cow when it finally passes the threshold? Is maybe the highly adapted cow rather a cow that adjusts DMY ‘in time’, i.e. at a lower threshold, but with less decrease in DMY? To be able to answer the question which cow is most valuable for the farmer in Rwanda, also the cows’ ability to stay healthy and to become and stay pregnant should be studied. Ravagnolo and Misztal (2002) used the THI on the day of insemination and found that the heritability for non-return rate increased with increasing THI. The genetic correlation between heat tolerance for non-return rate and heat tolerance for milk yield was close to zero, indicating the need to select for both these traits.

Breeding values for heat tolerance are not reported for Holstein–Friesian semen on the market today but Holstein–Friesian cattle can be selected for increased heat tolerance, as shown in Australia (Nguyen et al. 2017). Garner et al. (2016) used historical data from the milk recording scheme in the USA to identify cows with high and low breeding values for heat tolerance. A genomic evaluation of milk yield decline during heat waves was performed. Then, heat tolerant (HT) and heat susceptible (HS) cows were compared during a heat challenge experiment. The decline in daily milk yield from the baseline period was less for the HT cows (12% decline from baseline) than for the HS cows (17% decline from baseline).

We recommend that Ankole is used for systematic crossbreeding with Holstein–Friesian for a higher milk yield across seasons in Rwanda. For this AF cross breeding program, we recommend that Holstein–Friesian selected for heat tolerance are chosen when they become available on the global market. It should be remembered that a systematic cross with Ankole requires someone to take the responsibility for conserving and developing this indigenous breed. The percent of Ankole needed in crossbred cows to maintain the low effect of high THI found in this study is an area for future research.

## Data Availability

Data will be available on reasonable request.

## References

[CR1] Baumgard, L. and Rhoads, R.P., 2012. Effects of environment on metabolism. In: R.J. Collier and J.L. Collier (eds.), Environmental physiology of livestock, (John Wiley & Sons, West Sussex, UK), 81--100

[CR2] Berman A (2011). Invited review: are adaptations present to support dairy cattle productivity in warm climates?. Journal of Animal Science.

[CR3] Bernabucci U, Biffani S, Buggiotti L, Vitali A, Lacetera N, Nardone A (2014). The effects of heat stress in Italian Holstein dairy cattle. Journal of Dairy Science.

[CR4] Bouraoui R, Lahmar M, Abdessalem M, Djemali M, Belyea R (2002). The relationship of temperature-humidity index with milk production of dairy cows in a Mediterranean climate. Animal Research.

[CR5] Carabaño M, Bachagha K, Ramón M, Díaz C (2014). Modeling heat stress effect on Holstein cows under hot and dry conditions: selection tools. Journal of Dairy Science.

[CR6] Cowley FC, Barber DG, Houlihan AV, Poppi DP (2015). Immediate and residual effects of heat stress and restricted intake on milk protein and casein composition and energy metabolism. Journal of Dairy Science.

[CR7] Dado-Senn B, Vega Acosta L, Torres Rivera M, Field SL, Marrero MG, Davidson BD, Tao S, Fabris TF, Ortiz-Colón G, Dahl GE, Laporta J (2020). Pre- and postnatal heat stress abatement affects dairy calf thermoregulation and performance. Journal of Dairy Science.

[CR8] Dash S, Chakravarty AK, Singh A, Upadhyay A, Singh M, Yousuf S (2016). Effect of heat stress on reproductive performances of dairy cattle and buffaloes: A review. Veterinary World.

[CR9] De Rensis F, Garcia-Ispierto I, López-Gatius F (2015). Seasonal heat stress: Clinical implications and hormone treatments for the fertility of dairy cows. Theriogenology.

[CR10] Fernández IG, Ulloa-Arvizu R, Jorge Fernández J (2019). Milk yield did not decrease in large herds of high-producing Holstein cows in semi-arid climate of Mexico. Tropical Animal Health and Production.

[CR11] Garner JB, Douglas ML, Williams SRO, Wales WJ, Marett LC, Nguyen TTT, Reich CM, Hayes BJ (2016). Genomic selection improves heat tolerance in dairy cattle. Scientific Reports.

[CR12] Hahn G, Gaughan J, Mader T, Eigenberg R, De Shazer A (2009). Thermal indices and their applications for livestock environments. Livestock energetics and thermal environmental management.

[CR13] Herbut P, Angrecka S, Walczak J (2018). Environmental parameters to assessing of heat stress in dairy cattle – a review. International Journal of Biometeorology.

[CR14] Kadzere C, Murphy M, Silanikove N, Maltz E (2002). Heat stress in lactating dairy cows: a review. Livestock Production Science.

[CR15] Kekana TW, Nherera-Chokuda FV, Muya MC, Manyama KM, Lehloenya KC (2018). Milk production and blood metabolites of dairy cattle as influenced by thermal-humidity index. Tropical Animal Health and Production.

[CR16] Manzi M, Rydhmer L, Ntawubizi M, D’Andre Hirwa C, Karege C, Strandberg E (2020). Milk production and lactation length in Ankole cattle and Ankole crossbreds in Rwanda. Tropical Animal Health and Production.

[CR17] Manzi M, Rydhmer L, Ntawubizi M, Karege C, Strandberg E (2018). Growth traits of crossbreds of Ankole with Brown Swiss, Holstein Friesian, Jersey, and Sahiwal cattle in Rwanda. Tropical Animal Health and Production.

[CR18] Mugabo R., Nsabimana, J.D., Tuyisenge, J., Dusengemungu, L., Badege, P., Nyirigira, A., Nyirahorana, C. and Ngabo, M.G., 2019. Girinka Program as part of poverty reduction strategy in Rwanda: Ten Years Socioeconomic Impacts, (Rwanda Agriculture and Animal Resources Development Board (RAB), Kigali, Rwanda)

[CR19] National Research Council (1971). A guide to environmental research on animals.

[CR20] Nguyen TT, Bowman PJ, Haile-Mariam M, Nieuwhof GJ, Hayes BJ, Pryce JE (2017). Short communication: Implementation of a breeding value for heat tolerance in Australian dairy cattle. Journal of Dairy Science.

[CR21] Ravagnolo O, Misztal I, Hoogenboom G (2000). Genetic component of heat stress in dairy cattle, development of heat index function. Journal of Dairy Science.

[CR22] Ravagnolo O, Misztal I (2002). Effect of heat stress on nonreturn rate in Holstein cows: Genetic analyses. Journal of Dairy Science.

[CR23] Santana ML, Bignardi AB, Pereira RJ, Faro AMB (2016). Random regression models to account for the effect of genotype by environment interaction due to heat stress on the milk yield of Holstein cows under tropical conditions. Animal Genetics.

[CR24] Santana MLJ, Pereira RJ, Bignardi AB, Filho AE, Menéndez-Buxadera A, El Faro L (2015). Detrimental effect of selection for milk yield on genetic tolerance to heat stress in purebred Zebu cattle: Genetic parameters and trends. Journal of Dairy Science.

[CR25] SAS Institute Inc., 2017. SAS/STAT® 14.3 User’s Guide. Cary, NC: SAS Institute Inc

[CR26] Sejian V, Maurya VP, Kumar K, Naqvi SMK (2012). Effect of multiple stresses on growth and adaptive capability of Malpura ewes under semi-arid tropical environment. Tropical Animal Health and Production.

[CR27] Ter Steeg, I., 2019. Investment opportunities in the Rwandan dairy sector (TRAIDE Resilience Rwanda). (Report from TRAIDE Rwanda, available at www.agroberichtenbuitenland.nl)

[CR28] Wurzinger M, Mirkena T, Sölkner J (2014). Animal breeding strategies in Africa: Current issues and the way forward. Journal of Animal Breeding and Genetics.

